# Behavioural phenotypes of autism in autistic and nonautistic gender clinic-referred youth and their caregivers

**DOI:** 10.1177/13623613251379920

**Published:** 2025-10-30

**Authors:** Aimilia Kallitsounaki, Matthew C Fysh, David M Williams, Lauren Spinner, Eilis Kennedy

**Affiliations:** 1 University of Kent, UK; 2 Tavistock and Portman NHS Foundation Trust, UK; 3 University College London, UK

**Keywords:** ASD, autism traits, familial aggregation of autism, gender diversity, gender referrals in youth

## Abstract

In recent years, referrals of youth to specialised gender services have risen sharply, with ~11% of these youth diagnosed as autistic compared with a general population rate of ~1%. In two preregistered studies, we addressed this insufficiently understood intersection. In Study 1, we examined the number and developmental trajectory of autism traits in autistic and nonautistic gender clinic-referred and cisgender youth (aged 7–16 years) using both screening measures (Autism-Spectrum Quotient Children’s Version and Autism-Spectrum Quotient Adolescent Version, Social Communication Questionnaire–Lifetime) and diagnostic tools (Autism Diagnostic Interview–Revised, Brief Observation of Symptoms of Autism). In Study 2, we examined autism traits among the caregivers of participants from each group using the Autism-Spectrum Quotient Adolescent Version. Study 1 results showed the autism phenotype in autistic gender clinic-referred youth closely resembled that of their cisgender autistic peers. In addition, after addressing methodological limitations in previous research, we found no evidence of elevated autism traits in nonautistic gender clinic-referred youth, challenging findings of some earlier studies. Study 2 provided evidence of familial aggregation of both autism traits and diagnoses among caregivers of both autistic gender clinic-referred and cisgender participants. Taken together, these findings challenge the hypothesis that autism in gender-diverse youth is merely a ‘phenomimic’ of autism and provide valuable clinical insights into the presentation of autism in this population.

**Lay abstract:**

In recent years, more young people have been referred to specialised gender clinics for support with their gender identity. Interestingly, about 11% of these young people are also diagnosed with autism–much higher than the general population rate of only around 1%. This overlap has raised important questions about how autism and gender diversity are related, and even whether autism traits in gender-diverse people with an autism diagnosis really reflect autism. In this research, we carried out two studies to understand this link better. In the first study, we looked at autism traits in gender-diverse children and adolescents aged 7–16 years who were referred to a gender clinic for issues with their gender identity. We compared them with both autistic and nonautistic children who were not referred to gender services. We used several standard tools to assess autism-related traits, including both questionnaires and clinical interviews. We found that gender-diverse youth who were also autistic showed similar patterns of autism traits as cisgender autistic children who were not exploring their gender. Importantly, nonautistic gender-diverse youth did not show unusually high levels of autism traits, which challenges some earlier studies that suggested they might. In the second study, we explored autism traits in the caregivers (mostly mothers) of the young people in our first study. We found that caregivers of autistic children–whether the children were gender-diverse or not–were more likely to be autistic than the caregivers of nonautistic children. Moreover, even *non*autistic caregivers of autistic children displayed more autism traits than caregivers of nonautistic children, irrespective of whether their child was gender-diverse or not. Overall, our findings challenge the idea that autism in gender-diverse youth is just a mimic of ‘true’ autism caused by gender-related stress or experiences. Instead, the results point to genuine autism that presents in a typical way. This research provides important insights for clinicians and families, and highlights the need to take the gender-related concerns of autistic children as seriously as of nonautistic children.

Referrals to specialised gender clinics for gender-related concerns, including gender dysphoria, have risen considerably in recent years (e.g. Butler et al., 2018; de Graaf et al., 2018). Many of these gender clinic-referred youth experience co-occurring mental health difficulties or neurodevelopmental conditions, with autism spectrum disorder (ASD) being particularly prevalent (e.g. Chen et al., 2016; De Vries et al., 2010; Nyquist et al., 2024).

Autism is diagnosed on the basis of persistent social-communication difficulties and restricted, repetitive patterns of behaviour and interests (RRBIs) (American Psychiatric Association [APA], 2022). It is a highly heritable condition that runs in families (see Tick et al., 2016), with even nonautistic family members often exhibiting elevated autism traits (the ‘broad autism phenotype’; Bolton et al., 1994; Piven et al., 1997). While the global prevalence of autism is approximately 1% (Zeidan et al., 2022), the prevalence of ASD diagnoses among gender clinic–referred youth is around 11% (Kallitsounaki & Williams, 2023).

Research using parent report measures has also shown an increased number of autism traits in gender clinic–referred children and adolescents compared with the general population (Akgül et al., 2018; David et al., 2025; Leef et al., 2019; Mahfouda et al., 2019; Skagerberg et al., 2015; van der Miesen et al., 2018; VanderLaan et al., 2015). However, none of these studies assessed autism traits without accounting for diagnosed or under assessment cases in their samples. Given that autistic people are overrepresented in populations of gender clinic–referred children and adolescents, it is almost certain that samples of gender-diverse youth in the studies cited above contained autistic participants. This would have artificially inflated the average number of autism traits in those samples and could have driven the between-group differences in average number of autism traits.

Another key question concerns the developmental continuity and stability of autism traits in gender clinic–referred youth. Many prior studies rely on parent report measures asking whether a child currently or previously exhibited autism traits, making it unclear whether these traits remain stable over time. The present study addresses this gap by exploring both early and current autism traits across different groups.

A wider concern regards the nature of the autism traits in gender clinic–referred youth. Some have argued that minority stress and adverse social experiences among gender-diverse people can lead them to display clinical features that *resemble* autism but are not indicative of true ASD (Fortunato et al., 2022; Turban, 2018; Turban & van Schalkwyk, 2018). This is a major claim that requires careful unpacking. One understanding of this claim is that autism screening tools used in many studies lack sufficient diagnostic specificity. Hence, when elevated autism traits on these measures are reported in gender-diverse youth, these traits reflect psychological conditions other than autism (e.g. depression or anxiety). For example, Turban and van Schalkwyk (2018, p. 8) argue,

It is increasingly understood that such screening instruments can be nonspecific for ASD, with youth suffering from other emotional or behavioral problems having higher scores in the absence of ASD. Higher scores would be expected in youth with GD, because this population is known to have high rates of internalizing psychopathology.

For this reason, Turban and van Schalkwyk (2018) called for studies exploring the potential co-occurrence of autism and gender diversity to explore developmental trajectories and multiple informants/measures of autism features. They also called for studies of the overlap between gender diversity and autism that employ appropriate control groups to rule out explanations that focus on ascertainment biases and the risk of type 1 error in small samples. Our study is a response to this call.

A different way to understand this claim is that autism screening measures detect autistic traits, rather than internalising symptoms, in gender-diverse children, but these traits have a different developmental origin to those manifested by cisgender autistic children. Specifically, Turban and van Schalkwyk (2018) and Fortunato et al. (2022) argued that autism features may develop in gender-diverse youth as a downstream developmental consequence of minority stress and stigmatisation, and related anxiety and depression, rather than because of a biologically based form of neurodiversity:

. . .[elevated ASD traits in gender diverse youth] may be better understood not as signs of neurodiversity, but rather as the expression of specific issues of the discomfort related to GD. Specifically, social isolation, difficulties in their relationship with peers, repetitive patterns of behavior, intense fixations, and behavioral rigidity could be features of GD due to the deep discomfort and intense suffering that this condition may cause. We hypothesize that those aspects could be the result of individual defenses, which are displayed to cope. . . (Fortunato et al. 2022, p. 1213)

If this hypothesis is correct, several predictions follow. We tested each of these across two studies involving autistic gender-diverse youth, autistic cisgender youth, nonautistic gender clinic–referred youth, and nonautistic cisgender youth (Study 1) and their caregivers (Study 2).

First, in-depth clinical interviews and observational measures may not reveal the same degree of social-communication difficulties and RRBIs in gender clinic–referred autistic youth as suggested by screening measures, supporting the idea that screening tools lack specificity. Therefore, we assessed autism features among youth in Study 1 using in-depth diagnostic measures, as well as parent report (screening) questionnaires, with the aim to ensure that elevated scores on the screening measures did not reflect a lack of diagnostic specificity (Aim 1).

Second, autism traits may emerge later in development – rather than early in life – among gender clinic–referred youth (autistic and/or nonautistic), as minority stress is likely to manifest only after exposure to adverse social experiences. To explore this, we examined the presence and nature of autism features both currently and during early childhood, focusing on the critical developmental window around ages 4 to 5, when core autism traits typically become most apparent. Therefore, the second aim of Study 1 was to test the prediction stemming from the phenomimic theory that early features would be less pronounced (or possibly absent) than later features in gender clinic–referred youth (Aim 2).

Third, the familial transmission pattern of autism traits in gender clinic–referred autistic youth may differ from that seen in cisgender autistic individuals. If minority stress, rather than neurodevelopmental differences, underlies the presence of autism traits in gender clinic–referred youth, then their caregivers should not show the same elevation in autism traits observed in families of cisgender autistic children. Therefore, in Study 2, we assessed self-reported diagnoses of ASD and current number of ASD features manifested by the caregivers (mostly females) of youth who participated in Study 1. The aim was to determine whether the pattern of familial transmission of autism traits differs between gender clinic–referred and cisgender autistic youth, which would support or challenge the phenomimicry hypothesis (Aim 3).

## Study 1: method

### Participants

Participants were youth aged 7 to 16 years, assigned into four groups: autistic gender-referred, nonautistic gender-referred, autistic cisgender, and nonautistic cisgender (*N* = 259). Those with missing data, IQ below 70, or nongender clinic–referred children who expressed gender diversity were excluded from analyses (*n* = 30). Final sample characteristics are reported in Table 1. Groups were equated for assigned sex at birth (all *p*s ⩾ 0.411, BF_10_ = 0.09), but differed significantly in age, and full scale IQ (FSIQ) measured by the Vocabulary and Matrix Reasoning subtests of Wechsler Abbreviated Scale of Intelligence–II (WASI-II) (Wechsler, 2011). Given the need to maximise statistical power, unmatched samples were analysed and reported in this article. However, matched-sample analyses showed substantively identical results (i.e. no significant effect in the unmatched samples became nonsignificant in the matched samples, or vice versa) and are reported in Supplementary Information.

**Table 1.** Sample characteristics and two-way ANOVA statistics for age and FSIQ in Study 1.

Variable	Autistic		Nonautistic		ANOVA					Direction of effects
	GC-referred *n* = 49 (49% AFAB)	Cisgender *n* = 57 (44% AFAB)	GC-referred *n* = 56 (46% AFAB)	Cisgender *n* = 67 (52 % AFAB)	Effect	*F*	*p*	η_p_^2^	BF_10_	
	*M* (*SD*)	*M* (*SD*)	*M* (*SD*)	*M* (*SD*)						
Age	13.00 (2.35)	11.46 (2.11)	11.84 (2.17)	11.18 (2.42)	D	5.67	0.018	0.03	1.49	Autistic > Nonautistic
					G	13.33	<0.001	0.06	45.88	GC-referred > Cisgender
					D × G	2.14	0.145	0.01	0.53	
FSIQ	104.31 (13.91)	102.89 (15.11)	102.21 (11.78)	109.70 (12.06)	D	1.80	0.181	0.01	0.44	Autistic = Nonautistic
					G	2.99	0.085	0.01	0.79	GC-referred = Cisgender
					D × G	6.41	0.012	0.03	3.77	Cisgender: Autistic < Nonautistic
										GC-referred: Autistic = Nonautistic
										Autistic: Cisgender = GC-referred
										Nonautistic: Cisgender > GC-referred
Voc (*t* score)	53.08 (8.91)	51.60 (9.53)	52.66 (8.42)	57.30 (7.64)	D	5.33	0.022	0.02	2.72	Autistic < Nonautistic
					G	1.90	0.170	0.01	0.45	GC-referred = Cisgender
					D × G	7.16	0.008	0.03	5.14	Cisgender: Autistic < Nonautistic
										GC-referred: Autistic = Nonautistic
										Autistic: Cisgender = GC-referred
										Nonautistic: Cisgender > GC-referred
MR (*t* score)	51.94 (8.89)	51.82 (10.76)	50.09 (8.97)	53.99 (9.22)	D	0.02	0.902	0.00	0.15	Autistic = Nonautistic
					G	2.24	0.136	0.01	0.49	GC-referred = Cisgender
					D × G	2.52	0.114	0.01	0.66	

*Note. N* = 229. AFAB = assigned female at birth; ANOVA = analysis of variance; FSIQ = full scale IQ-2; Voc = vocabulary subtest; MR = matrix reasoning subtest; GC-referred = gender clinic–referred; D = diagnostic status; G = gender identity status.

Cisgender participants were recruited through schools, social media, autism charities, and the Kent Child Development Unit database. Gender clinic–referred participants had been referred to a specialist clinic, and were recruited from an ongoing prospective longitudinal study (Kennedy et al., 2021) and social media. Out of 105 gender clinic–referred participants, 100 reported a gender that did not align with their assigned sex at birth, with the remaining five reporting congruence between gender and assigned sex at birth. Excluding these five participants did not change results (see Supplementary Information), so they were retained in the main analyses. Almost all participants (99.13%) were native English speakers. Information about participants’ ethnicity/race is presented in Table 2.

**Table 2.** Descriptive statistics for race/ethnicity in Study 1.

Ethnicity/Race	Autistic		Nonautistic	
	GC-referred	Cisgender	GC-referred	Cisgender
	*n* (%)	*n* (%)	*n* (%)	*n* (%)
African	0 (0)	0 (0)	1 (1.79)	0 (0)
Asian	0 (0)	0 (0)	0 (0)	1 (1.49)
British	45 (91.84)	51 (89.47)	45 (80.36)	53 (79.10)
Other White	0 (0)	1 (1.75)	5 (8.93)	8 (11.94)
White and Asian	0 (0)	2 (3.51)	4 (7.14)	1 (1.49)
White and Black African	1 (2.04)	0 (0)	0 (0)	2 (2.99)
White and Black Caribbean	1 (2.04)	2 (3.51)	0 (0)	0 (0)
Other multi-ethnic groups	2 (4.08)	1 (1.75)	1 (1.79)	2 (2.99)

All participants in the autism groups had a formal ASD diagnosis (*M*_age of diagnosis_ = 8.61, *SD* = 2.97, *n* = 104) except for eight participants who were undergoing assessment. Excluding these participants did not change results (see Supplementary Information), so they were retained in the main analyses. To verify diagnoses, caregivers were asked to present the diagnostic letter during a virtual session with a researcher. They also provided written information regarding the type of diagnosis, the date it was received, and the type of professional (child psychiatrist, paediatrician, etc.) who made the diagnosis.

All participants completed the study after providing electronic informed consent or assent. The study was approved by the Kent Psychology (approval number: 202216553002907588) and HRA and London–Hampstead (reference number: 22/LO/0805) Research Ethics Committees. The study was preregistered on Open Science Framework (preregistration can be viewed here https://osf.io/hfgd2/?view_only=69290591d7e44640ad8765d8c6f1f5ad; note the file/folder needs to be downloaded). Deviations from the preregistration are presented in the Supplementary Information.

### Materials

#### Early autism traits

##### Autism Diagnostic Interview – Revised

The ADI-R (Rutter et al., 2003) is a standardised, comprehensive diagnostic interview conducted with caregivers to assess their child’s early development and current behaviour. It comprises 93 items covering four domains: (a) Reciprocal Social Interactions; (b) Communication; (c) Restricted, Repetitive, and Stereotyped Patterns of Behaviour; and (d) Atypicality of Development Evident at or Before 36 Months. For the purposes of this study, only the 42 items included in the official scoring algorithm were administered and scored (16 items in Domain A, 13 in Domain B, eight in Domain C, and five in Domain D). Items in Domains A, B, and C are each rated on a scale from 0 to 2, with higher scores indicating greater ASD feature severity (note that scores of 3 are possible, but are conventionally recoded as 2). Items in Domain D are scored on a scale from 0 to 1. Scores for each domain are summed to reflect either the severity of autism features in Domains A, B, and C, or the presence or absence of each trait in Domain D. Cutoff scores that indicate clinically significant features consistent with an ASD diagnosis are 10 for Domain A (Reciprocal Social Interaction), 8 (or 7 for nonverbal children) for Domain B (Communication), and 3 for Domain C (Restricted and Repetitive Behaviours).

It is important to be clear that 20 of the 42 items in the scoring algorithm probe for ASD features manifested during the developmental window of 4 and 5 years, with the remainder probing for ASD features manifested at any point in the child’s life. The 20 items that specifically concern behaviour in the 4- to 5-year age window are all from Domains A (13 items) and B (seven items). In addition to analysing the 42 items in the standard manner, we also employed a second, more targeted scoring approach to focus specifically on early-emerging autism features. For this additional analysis, only the 13 items from Domain A and the seven items from Domain B that assess behaviour during the 4- to 5-year age window were selected. Scores on these items were dichotomised, such that a score of zero indicated the absence of an ASD feature and any nonzero score (i.e. a score of 1 or 2) was recoded as 1, indicating the presence of an ASD feature. A composite binary score was then computed to reflect the overall presence or absence of early social-communication difficulties. Note that only caregivers of autistic participants completed this measure.

Interrater reliability was established on 80% of assessments and was excellent (Cicchetti, 1994) in every domain (ICC: 0.90–0.95). The ADI-R has an overall estimated sensitivity of 0.75 and a specificity of 0.82 (Lebersfeld et al., 2021).

##### Social Communication Questionnaire–Lifetime

The Social Communication Questionnaire (SCQ)–Lifetime (Rutter et al., 2003) is a widely used parent report questionnaire consisting of 40 yes/no questions designed to tap autism traits in youth. Only 21 items assessing early development (ages 4–5) were analysed. Higher scores indicate more autism traits.

#### Current autism traits

##### Brief Observation of Symptoms of Autism

The Brief Observation of Symptoms of Autism (BOSA) (Dow et al., 2022; Lord et al., 2020), a 12- to 16-min structured caregiver–child interaction recorded via MS Teams or Zoom, was completed by autistic youth aged 7 to 10 (BOSA-F1) and 11 to 16 years (BOSA-F2). To ensure consistency and comparability across participants, the Autism Diagnostic Observation Schedule, Second Edition (ADOS-2) Module 3 protocol was used for all participants, including those over 15 years of age. ADOS-2 codes were transferred to the BOSA *DSM*-5 checklist, which is designed to align observed behaviour with *DSM*-5 criteria for ASD, and then converted to binary values to indicate the presence (1) or absence (0) of each trait. Domain A assesses Social-Communication and -Interaction (scores of 0–10) and Domain B assesses Restricted and Repetitive Behaviours (scores of 0–5). Note that only autistic participants completed this measure.

Total scores range from 0 to 15, with higher scores denoting a greater number of autism traits. A cut-off score of 6 indicates clinically significant autism traits. Eighty percent of assessments were scored by a second ADOS-2 trained rater. Interrater reliability was 0.85 (intraclass correlation coefficients (ICCs): Domain A (Social-Communication and -Interaction) = 0.80; Domain B (Restricted and Repetitive Behaviours) = 0.88), which is excellent according to Cicchetti’s (1994) criteria.^1^ The BOSA has demonstrated excellent test–retest reliability (ICC = 0.95), high discrimination ability (area under the curve (AUC) for Module 3 = 0.91), and strong convergent validity with the ADOS-2 (Module 3 Overall Total: *r* = 0.63; Dow et al., 2022).^2^

##### Autism-Spectrum Quotient

The Autism Spectrum Quotient Children’s Version (AQ-Child)/Autism Spectrum Quotient Adolescent Version (AQ-Adolescent) (Auyeung et al., 2008; Baron-Cohen et al., 2006) are 50-item parent report questionnaires, rated on a 4-point Likert-type scale ranging from *definitely agree* to *definitely disagree*, about autism traits in children. The AQ-child was completed about participants aged 7 to 11 years, and the AQ-adolescent was completed about participants aged 12 to 16 years. Responses were converted to binary scores, which range from 0 to 50, with higher scores indicating more autism traits. Both versions have demonstrated good to excellent test–retest reliability (⩾ .85), moderate to high internal consistency (Cronbach’s α ⩾ 0.79), and acceptable to strong discrimination ability (Auyeung et al., 2008; Baron-Cohen et al., 2006). They have also shown convergent validity with another parent report measure, the Social Responsiveness Scale (*r* = 0.64; Armstrong & Iarocci, 2013).

### Procedure

Most participants completed the Matrix Reasoning and Vocabulary subtests of the WASI-II, as well as the BOSA, online via a virtual session with a researcher. However, one participant completed the BOSA and the WASI-II in person in a lab-based setting, and nine participants completed the WASI independently via a Qualtrics link. For those who completed the WASI independently, instructions identical to those provided to the other participants were given. In addition, it was stressed to caregivers that the child should not use any dictionaries or seek help while completing the task. Caregivers completed all parent report questionnaires independently via a Qualtrics link and completed the ADI-R via an online virtual session with a researcher.

### Statistical analysis

Effect sizes were quantified using partial eta squared (values of 0.01, 0.06, 0.14 represent small, medium, and large effects, respectively), Cohen’s *d* (0.20, 0.50, 0.80 representing small, medium, and large effects), and Phi (φ) (0.10, 0.30, 0.50 representing small, medium, and large effects). Bayesian analyses were also performed to assess the relative strength of the alternative hypothesis compared with the null (e.g. Dienes, 2014). Bayes factors (BF_10_ > 1) indicate increasing support for the alternative hypothesis (BF_10_ > 3, >10, and >30 represent substantial, strong, and very strong evidence for H1, respectively), whereas those < 1 provide increasing evidence in favour of the null hypothesis (BF_10_ < 0.33, <0.10, and <0.03 represent substantial, strong, and very strong evidence for H0, respectively). All Bayesian analyses were performed using R (version 4.3.3; R Core Team, 2024) and JASP 0.19.3 (JASP team, 2025).

## Study 1: results

### Developmental trajectories of autism traits on screening and diagnostic measures

**Figure 1.**
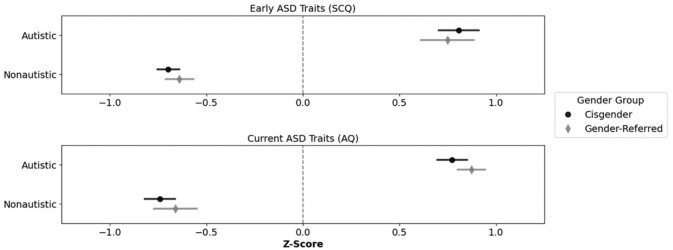
Mean standardised scores of autism traits by diagnostic status and gender identity over time. *Note.* SCQ = Social Communication Questionnaire; AQ = Autism-Spectrum Quotient.

Figure 1 shows the z-transformed SCQ and AQ scores, reflecting early and current autism traits, respectively, among youth participants. A 2 (diagnostic status: autistic/nonautistic) × 2 (gender identity status: gender clinic-referred/cisgender) × 2 (developmental timepoint: early/current) mixed analysis of variance (ANOVA) on these data revealed a significant main effect of diagnostic status, *F*(1, 225) = 379.29, *p* < 0.001, η_p_^2^ = 0.63, BF_10_ > 100, reflecting significantly higher autism traits among autistic (*M* = 0.80, *SD* = 0.62) than nonautistic youth (*M* = −0.69, *SD* = 0.56). Neither the main effect of developmental timepoint, *F*(1, 225) = 0.02, *p* < 0.896, η_p_^2^ < 0.01, BF_10_ = 0.10), nor the main effect of gender identity status, *F*(1, 225) = 0.34, *p* = 0.559, η_p_^2^ < 0.01, BF_10_ = 0.28, was significant. All interactions were also nonsignificant (*p*s ⩾ 0.339, η_p_^2^ < 0.01, BF_10_s ⩽ 0.28). Thus, regardless of their gender identity status, autistic participants demonstrated significantly more autism traits (both early and current) on screening/questionnaire measures than nonautistic participants.

Table 3 shows the early and current social-communication features *in autistic participants only* as measured using diagnostic tools. These scores were derived from a composite of ADI-R Domains A (Reciprocal Social Interactions) and B (Communication) to measure early social-communication features, and BOSA Domain A (Social Communication and Interaction) to measure current social-communication features. Note that only the ADI-R items probing social-communication features in the 4- to 5-year age window were used in this analysis. Composite scores were then z-transformed and submitted to a 2 (developmental timepoint: early/current) × 2 (gender identity status: gender clinic-referred/cisgender) mixed ANOVA. No results were significant, indicating no differences in early or current social-communication features between autistic gender clinic–referred and autistic cisgender participants (note that only autistic participants completed the BOSA and ADI-R, so diagnostic status was not included as a factor in this analysis).

**Table 3.** Means, standard deviations, and two-way ANOVA statistics for autism traits assessed with diagnostic tools in Study 1.

Variable	Autistic		ANOVA					Direction of Effects
	GC-referred	Cisgender	Effect	*F*	*p*	η_p_2	BF_10_	
	*M* (*SD*)	*M* (*SD*)						
Autism Traits								
Early (ADI-R)	-0.09 (1.14)	0.07 (0.88)	G	0.26	0.614	0.00	0.20	GC-referred = Cisgender
Current (BOSA)	0.01 (0.97)	-0.01 (1.03)	T	0.01	0.936	0.00	0.15	Early = Current
			T × G	0.40	0.529	0.00	0.28	

*Note. N* = 94 (*n* = 41 for autistic gender clinic-referred group; *n* = 53 for autistic cisgender group). ANOVA = analysis of variance; GC-referred = gender clinic–referred; G = gender identity status; T = timepoint; ADI-R = Autism Diagnostic Interview–Revised; BOSA = Brief Observation of Symptoms of Autism.

### Emergence and presentation of autism features on diagnostic measures of *early and lifetime features*

In the autistic gender clinic–referred group, 34/45 participants (75.56%) scored above the cut-off on ADI-R Domain D (Atypicality of Development Evident at or Before 36 Months), compared with 49 out of 57 (85.96%) in the autistic cisgender group. A chi-square test revealed a small and nonsignificant between-group difference in the proportion of participants who manifested developmental atypicalities at or before the age of 3 years, χ^2^(1, *N* = 102) = 1.80, *p* = 0.180, ϕ = −0.13, BF_10_ = 0.46.

**Figure 2.**
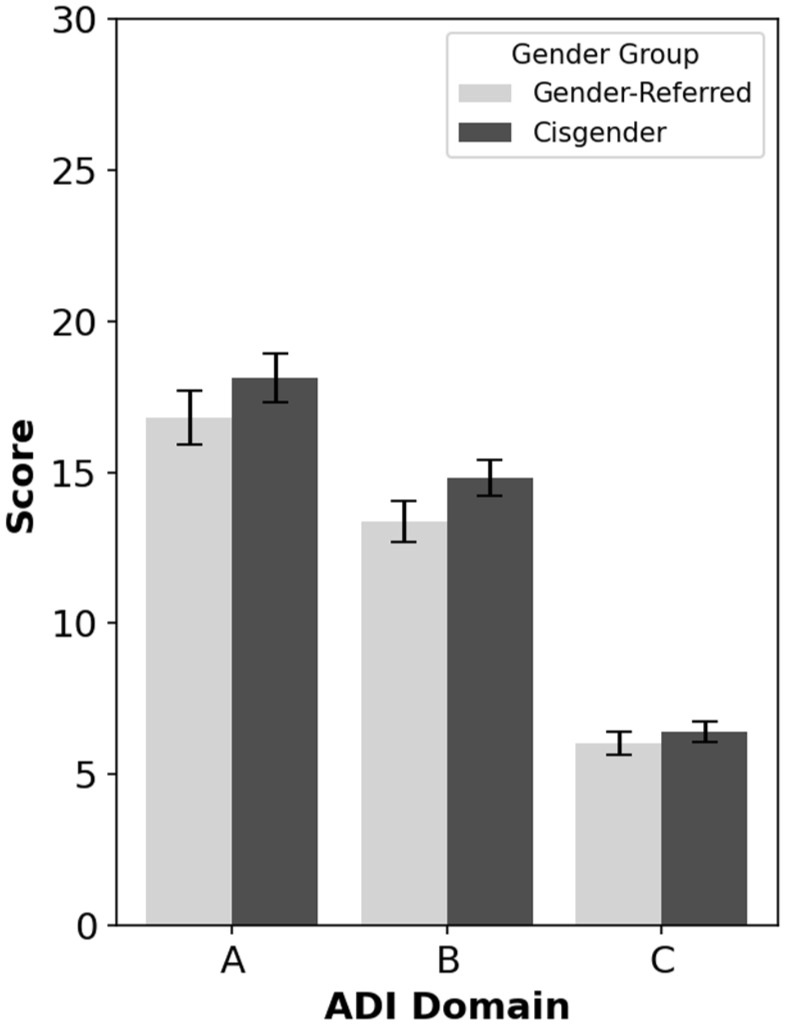
ADI-R scores for autistic gender clinic-referred and autistic cisgender participants across domains. *Note. N* = 102 (*n* = 45 for autistic gender clinic-referred group; *n* = 57 for autistic cisgender group). Domain A **=** Reciprocal Social Interactions; Domain B = Communication; Domain C = Restricted, Repetitive, and Stereotyped Patterns of Behaviour; GC-referred = gender clinic-referred. Error bars show standard errors.

Figure 2 shows mean scores in each autism group on Domains A (Reciprocal Social Interactions), B (Communication), and C (Restricted, Repetitive, and Stereotyped Patterns of Behaviour) of the ADI-R. Note that all ADI-R items from the scoring algorithm were included, not only those that targeted autism features in the 4- to 5-year age window. Multivariate analysis of variance (MANOVA) indicated no overall group differences across ADI-R domains, Pillai’s Trace = 0.02, *F*(3, 98) = 0.81, *p* = 0.494, BF_10_ = 0.06. Thus, cisgender and gender clinic–referred autistic participants did not differ significantly in severity of lifetime reciprocal social interaction features, communication features, or restricted, repetitive, and stereotyped interests and behaviours.

#### Current features

BOSA total score and BOSA Domain B (Restricted and Repetitive Behaviours) scores were analysed using independent *t* tests (note that BOSA Domain A (Social-Communication and -Interaction) score was analysed above and revealed no significant difference between autistic gender-diverse and autistic cisgender participants). There was a nonsignificant difference between autistic gender clinic–referred (*n* = 41, *M* = 8.49, *SD* = 2.79) and autistic cisgender (*n* = 53, *M* = 8.98, *SD* = 2.76) participants in the BOSA total score, *t*(92) = −0.85, *p* = 0.395, *d* = 0.18, BF_10_ = 0.30. However, autistic gender clinic–referred participants showed fewer current restricted interests and behaviours on Domain B (Restricted and Repetitive Behaviours) (*n* = 41, *M* = 1.71, *SD* = 1.21) than autistic cisgender participants (*n* = 53, *M* = 2.25, *SD* = 1.13), *t*(92) = −2.23, *p* = 0.029, *d* = 0.46, BF_10_ = 1.88.

## Study 1: discussion

Results suggested that the autism phenotype in gender clinic–referred autistic youth is very similar to that manifested by cisgender autistic youth. Parental questionnaire measures showed that early and current autism traits were similar between gender clinic–referred autistic participants and cisgender autistic participants. Both groups showed significantly elevated scores relative to nonautistic gender clinic–referred and nonautistic cisgender children at both time periods. Crucially, in-depth interview (ADI-R) and observational (BOSA) measures confirmed results from parental questionnaires, with no significant differences observed between cisgender autistic children and gender clinic–referred autistic children in either early or current autism features, except in one respect. Domain B (Restricted and Repetitive Behaviours) scores on the BOSA implied that repetitive behaviours and interests were less observable among gender clinic–referred autistic participants than cisgender autistic participants. We return to possible explanations for this finding in the general discussion. In Study 2, we investigated the number of self-reported autism traits in the caregivers of nonautistic gender clinic–referred, nonautistic cisgender, autistic gender clinic–referred, and autistic cisgender youth.

## Study 2: method

### Participants

Caregivers of autistic gender clinic–referred, nonautistic gender clinic-referred, autistic cisgender, and nonautistic cisgender children and young people participated in this study (*N* = 259). Participants with missing data on key variables, as well as those with nongender clinic–referred children who either expressed gender diversity or for whom child-report data on gender identity were not available, were excluded from analyses (*n* = 33). The final sample characteristics are reported in Table 4. Fisher’s exact tests indicated that the nonautistic cisgender group included significantly more assigned male at birth caregivers than the autistic gender clinic–referred group (*p* = 0.006, BF_10_ = 17.75). All other between-group differences in sex ratio were nonsignificant (all *p*s ⩾ 0.065, BF_10s_ ⩽ 0.78). In addition, groups were not matched for age (see Table 4). Note that the results of the main analyses did not change substantively when groups were matched for age and assigned sex at birth (see Supplementary Information).

**Table 4.** Sample characteristics and two-way ANOVA statistics for age and autism traits in Study 2.

Variable	Autistic		Nonautistic		ANOVA					
	GC-referred	Cisgender	GC-referred	Cisgender	Effect	*F*	*p*	η_p_^2^	BF_10_	Direction of effects
	*n* = 52 (98% AFAB)	*n* = 56 (93% AFAB)	*n* = 55 (91% AFAB)	*n* = 63 (81% AFAB)						
	*M* (*SD*)	*M* (*SD*)	*M* (*SD*)	*M* (*SD*)						
Age	45.13 (4.76)	42.61 (6.54)	44.47 (6.59)	43.19 (4.36)	D	0.00	0.958	0.00	0.15	Autistic = Nonautistic
					G	6.44	0.012	0.03	2.77	GC-referred > Cisgender
					D × G	0.69	0.408	0.00	0.26	
Autism traits (AQ-50)	20.33 (11.94)	21.73 (11.53)	14.82 (8.99)	15.59 (6.59)	D	19.60	<0.001	0.08	> 100	Autistic > Nonautistic
					G	0.68	0.410	0.00	0.19	GC-referred = Cisgender
					D × G	0.06	0.809	0.00	0.22	

*Note. N* = 226. AFAB = assigned female at birth; ANOVA = analysis of variance; GC-referred = gender clinic–referred; D = child diagnostic status; G = child gender identity status; AQ-50 = Autism-Spectrum Quotient.

Ninety-six percent of participants were biological parents. For 91.59% of participants, English was their first language. Information about participants’ ethnicity/race is presented in Table 5. Recruitment procedures followed those described in Study 1.

**Table 5.** Descriptive statistics for race/ethnicity in Study 2.

Ethnicity/Race	Autistic		Nonautistic	
	GC-referred	Cisgender	GC-referred	Cisgender
	*n* (%)	*n* (%)	*n* (%)	*n* (%)
African	0 (0)	0 (0)	1 (1.82)	0 (0)
Arab	1 (1.92)	0 (0)	0 (0)	0 (0)
British	47 (90.38)	48 (85.71)	46 (83.64)	48 (76.19)
Irish	0 (0)	0 (0)	1 (1.82)	1 (1.59)
Other White	2 (3.85)	5 (9.83)	6 (10.91)	11 (17.46)
Indian	0 (0)	0 (0)	1 (1.82)	0 (0)
Other Asian	0 (0)	0 (0)	0 (0)	2 (3.17)
Other ethnic group	1 (1.92)	0 (0)	0 (0)	0 (0)
White and Asian	0 (0)	2 (3.57)	0 (0)	0 (0)
White and Black Caribbean	1 (1.92)	0 (0)	0 (0)	0 (0)
Other multi-ethnic groups	0 (0)	1 (1.79)	0 (0)	1 (1.59)

### Materials and procedure

#### Autism diagnosis

As part of a self-report demographic questionnaire, caregivers were asked whether they had a formal diagnosis of autism.

#### Autism-Spectrum Quotient

The AQ-Adult (Baron-Cohen et al., 2001) is a 50-item self-report questionnaire, rated on a 4-point Likert-type scale ranging from *definitely agree* to *definitely disagree*, about autism traits in adults. Responses were converted to binary scores, which range from 0 to 50, with higher scores indicating more autism traits. The AQ-50 has shown good internal consistency (Cronbach’s α = 0.81; Wakabayashi et al., 2006) and convergent validity with the Social Responsiveness Scale (>0.55; Armstrong & Iarocci, 2013; Ingersoll et al., 2011). All caregivers completed the AQ-50 independently via a Qualtrics link.

## Study 2: results

### Autism diagnosis in caregivers

A series of one-proportion *z* tests compared the proportion of ASD diagnoses in each group of caregivers with the general population prevalence (1%). The results showed that the proportion of ASD diagnoses was significantly higher in caregivers of autistic gender clinic–referred (7.69%) and autistic cisgender (7.14%) youth than in the general population, *z* = 4.85, *p* < 0.001, BF_10_ = 17.85 and *z* = 4.62, *p* < 0.001, BF_10_ = 14.37, respectively. None of the caregivers in the nonautistic groups (gender clinic–referred or cisgender) reported a diagnosis of ASD, and this did not differ significantly from the population estimate, *z* = −0.75, *p* = 0.453, BF_10_ = 0.94 and *z* = −0.80, *p* = 0.424, BF_10_ = 0.95, respectively. Hence, ASD diagnoses were overrepresented in the caregivers of autistic youth, regardless of their children’s gender identity status, compared with the general population estimate.

### Autism traits in caregivers

A 2 (child diagnostic status: autistic/nonautistic) × 2 (child gender identity status: gender clinic-referred/cisgender) ANOVA was conducted on caregiver AQ-50 score to examine the effect of child’s gender identity status and diagnostic status on caregivers’ self-reported autism traits. This revealed a significant main effect of child diagnostic status, indicating that caregivers of autistic participants reported significantly more autism traits than caregivers of nonautistic children, regardless of their child’s gender identity status (see Table 5). Neither the main effect of child gender identity status nor the Child Gender Identity Status × Child Diagnostic Status interaction was significant. When caregivers with, or awaiting assessment for, a diagnosis of ASD were excluded, results did not change (see Supplementary Information).

## Study 2: discussion

In Study 2, categorical diagnoses of ASD, as well as number of autism traits, were significantly higher among the caregivers of autistic youth than nonautistic youth, regardless of the gender identity status of the youth. The diagnostic rate of autism was significantly above the population estimate of 1% in caregivers of autistic children, but not caregivers of nonautistic children, independent of youth gender identity.

Crucially, the effect of youth autism status on the number of caregiver autism traits remained significant after excluding caregivers with a diagnosis of ASD. This is important because it shows that a broad autism phenotype is apparent in the nonaffected family members of both gender clinic–referred and cisgender autistic youth (e.g. Bolton et al., 1994; Piven et al., 1997). In contrast, the caregivers of nonautistic gender clinic–referred youth did not show significantly more autism traits than the caregivers of nonautistic cisgender youth, and none of the caregivers in either group reported a diagnosis of autism.

## General discussion

To our knowledge, this study provides the most comprehensive assessment to date of autism phenotypes in gender clinic–referred gender-diverse children. No previous study among gender clinic–referred children has (a) investigated the early manifestation of autism features (at the critical ages 4 to 5 years), (b) used both parent report questionnaires and gold standard diagnostic tools involving direct observation and in-depth parental interview, or (c) investigated autism features among caregivers.

Results suggested that the autism phenotype in gender clinic–referred children with an ASD diagnosis is very similar to that manifested by cisgender children with an ASD diagnosis. Parental questionnaires showed that early and current features were similarly elevated in both groups, a finding largely confirmed by in-depth diagnostic measures. The ADI-R revealed that autistic gender clinic–referred participants were as likely as autistic cisgender participants to exhibit autism traits before or at age 3, with both groups displaying equivalent features in the critical 4- to 5-year-old period. Similarly, the BOSA confirmed that social-communication features diagnostic of ASD were equally observable in gender clinic–referred and cisgender autistic children. These results show that the presentation and developmental pattern of autism features are largely equivalent in gender clinic–referred and cisgender autistic youth, and that screening measures tap genuine autism features in both sets of children.

These findings do not support the hypothesis that autism traits in gender-diverse autistic youth arise from minority stress rather than neurodiversity (Fortunato et al., 2022; Turban, 2018; Turban & van Schalkwyk, 2018). If minority stress caused a ‘quasi-autism’ in gender-diverse autistic children, we would expect to see elevated autism traits in gender clinic–referred nonautistic children compared with cisgender nonautistic children, given their exposure to minority stress (e.g. Hunter et al., 2021; Wilson et al., 2024). However, we did not observe this. This key finding contrasts with previous studies in gender clinic–referred youth (Akgül et al., 2018; David et al., 2025; Leef et al., 2019; Mahfouda et al., 2019; Skagerberg et al., 2015; van der Miesen et al., 2018; VanderLaan et al., 2015). Notably, this study was the first to ensure that the sample of nonautistic gender clinic–referred youth was well characterised, explicitly excluding children with ASD diagnoses or those under assessment, preventing an artificial inflation of autism trait scores in the group.

We also observed a significantly higher rate of ASD diagnoses among the caregivers of both cisgender and gender clinic–referred autistic children, relative to cisgender and gender clinic–referred nonautistic children. Even after diagnosed caregivers were excluded, the caregivers of autistic children showed significantly elevated autism traits relative to the caregivers of nonautistic children, irrespective of their child’s gender identity status. Importantly, ASD diagnoses and autism trait levels did not significantly differ between caregivers of gender clinic–referred nonautistic and cisgender nonautistic youth. This is the first study to show that autism is familial among gender clinic–referred youth, just as it is among cisgender youth. While this does not confirm a genetic basis for autism in either group, it supports the idea that autism has the same underlying origins across groups.

In sum, results from Studies 1 and 2 demonstrate that, as in cisgender autistic children, autism in gender clinic–referred autistic youth (a) manifests early in life (at or before age 3), (b) is identified through both screening and diagnostic tools, and (c) is elevated in caregivers. These findings do not support the claim that autism in gender-diverse youth is a phenomimic/quasi-autism. If minority stress, rather than neurodiversity, led to autism-like traits, it is unclear why these traits would appear before age 3, given the likely minimal exposure to minority stress at that stage. Even if one argued that minority stress affects children that young, it does not explain why caregivers of gender clinic-referred autistic children would experience enough stress to develop a quasi-autism. In addition, if such stress were present, it is unlikely to exceed the levels experienced by caregivers of gender clinic–referred nonautistic children. Thus, the lack of elevated autism traits in gender clinic–referred nonautistic children and their caregivers further weakens the phenomimicry hypothesis.

The only finding in the current paper that might be consistent with the phenomimic hypothesis was that gender clinic–referred autistic participants showed significantly fewer RRBIs on the BOSA than cisgender autistic participants. One explanation for this is that Domain B (Restricted and Repetitive Behaviours) of the BOSA is less sensitive to RRBIs than Domain A (Social-Communication and -Interaction) is to social-communication features. Notably, Domain B was the only subscale on either the BOSA or ADI-R that initially produced inter-rater reliability below an acceptable threshold and required raters to resolve disagreements. However, the between-group differences in Domain B scores remained significant after disagreements between raters were resolved. Moreover, the measure was sensitive enough to detect RRBIs in cisgender autistic participants, which suggests that the current results may not be artefactual. It is not clear what might cause a reliable difference between the two autism groups in current observable RRBIs, especially given that Domain C (Restricted, Repetitive, and Stereotyped Patterns of Behaviour) of the ADI-R, which taps lifetime restricted and repetitive interests and behaviours, did not show any significant differences between cisgender and gender clinic–referred autistic participants. In the context of all the other results of Studies 1 and 2, we do not think that the results from Domain B of the BOSA provide concrete evidence for the phenomimicry hypothesis, but we suggest further investigation of RRBIs in gender-diverse children and adolescents is warranted.

It is also worth noting that the findings from Studies 1 and 2 might not be fully generalisable to other populations. This, of course, is true of all studies, so is not a limitation, but rather a standard note of caution about extrapolating conclusions about an entire population based on data from a sample of that population. We do not know whether the same results would be observed in youth or caregivers with intellectual impairment for example. Likewise, the gender-diverse participants in this study were all clinic-referred. This ensured a clearly characterised sample with documented gender divergence and associated support needs, but it may not capture the full spectrum of gender diversity. Future research could usefully explore the autism phenotype in nonreferred samples of gender-diverse youth.

Finally, it is worth noting that the atypical, unstandardized administration of the WASI-II to nine of the 229 participants in Study 1 (who completed the subtests without an experimenter present) may have influenced their performance. Likewise, the BOSA (the objective measure of current ASD traits used in Study 1) is a brief, virtual adaptation of the ADOS-2, which may influence performance. There are fewer items in the BOSA than ADOS, so it captures fewer features and therefore nuances in profile. However, its shorter duration and more familiar setting may be preferable for some autistic children, reducing anxiety and enhancing ecological validity. More clearly, the BOSA demonstrates excellent psychometric properties, including strong convergent validity with the ADOS-2 and discriminant validity in identifying autistic people that seems at least as good as ADOS-2.

Overall, the current results suggest that clinicians should offer the same support for gender-diverse children who present with autism features as they would offer cisgender peers. If autism features in gender clinic–referred children were phenomimics, then clinicians might justifiably focus on supporting gender-related issues in the belief that autism features would reduce or disappear after gender issues were addressed. However, our results suggest this would be inappropriate because the autism features in gender clinic–referred youth are unlikely to be phenomimics. As such, it is imperative that gender-diverse children receive the same access to ASD-related services as cisgender children when ASD is suspected or diagnosed. To achieve this, awareness among clinicians and frontline professionals, such as general practitioners and school staff, should be increased through targeted training that addresses the intersection of autism and gender diversity. Given the rapid pace of research in this area, continuing professional development is crucial to ensure evidence-based practice is implemented.

## Supplemental Material

sj-docx-1-aut-10.1177_13623613251379920 – Supplemental material for Behavioural phenotypes of autism in autistic and nonautistic gender clinic-referred youth and their caregivers Supplemental material, sj-docx-1-aut-10.1177_13623613251379920 for Behavioural phenotypes of autism in autistic and nonautistic gender clinic-referred youth and their caregivers by Aimilia Kallitsounaki, Matthew C Fysh, David M Williams, Lauren Spinner and Eilis Kennedy in Autism

## Notes

1. The initial interrater reliability for Domain B fell below the threshold for ‘excellent’ according to Cicchetti’s (1994) criteria. As a result, the raters reviewed recordings in which their scores differed by more than one point. In cases where consensus could not be reached, a third rater, also trained in Autism Diagnostic Observation Schedule, Second Edition (ADOS-2), independently reviewed and resolved the discrepancy.

2. Researchers who administered and scored the diagnostic measures (Brief Observation of Symptoms of Autism (BOSA) and Autism Diagnostic Interview–Revised (ADI-R)), described below, completed certified training courses provided by a certified independent ADI-R and ADOS-2 trainer affiliated with the University of California, San Francisco. This ensured adherence to established research-reliability standards of administering, coding, and interpreting the measures.
