# Supplementary material for: Behavioural phenotypes of autism in autistic and nonautistic gender clinic-referred youth and their caregivers
Source: Autism. 2025 Oct 30;30(1):163–75. doi: 10.1177/13623613251379920 (PMC12717293; doi:10.1177/13623613251379920)
Supplement: sj-docx-1-aut-10.1177_13623613251379920 – Supplemental material for Behavioural phenotypes of autism in autistic and nonautistic gender clinic-referred youth and their caregivers [file sj-docx-1-aut-10.1177_13623613251379920.docx]

**Supplementary Information**

**Contents**

[1. Results After Matching Groups for Age and FSIQ in Study 1 2](#_Toc194675053)

[1.1 Developmental Trajectories of Autism Traits 2](#_Toc194675054)

[1.1.1 Matching Procedure and Results on SCQ and AQ. 2](#_Toc194675055)

[1.1.2 Matching Procedure and Results on ADI-R and BOSA. 6](#_Toc194675056)

[1.2 Emergence and Presentation of Autism Traits on Diagnostic Measures 8](#_Toc194675057)

[1.2.1 Matching Procedure and Results on ADI-R. 8](#_Toc194675058)

[1.2.2 Matching Procedure and Results on BOSA. 9](#_Toc194675059)

[2. Results After Excluding Participants who Identified With Their Assigned Sex at Birth From the Gender Clinic-Referred Group in Study 1 11](#_Toc194675060)

[2.1 Developmental Trajectories of Autism Traits on Screening and Diagnostic Measures 11](#_Toc194675061)

[2.2 Emergence and Presentation of Autism Traits on Diagnostic Measures 14](#_Toc194675062)

[2.2.1 Early and Lifetime Traits. 14](#_Toc194675063)

[2.2.2 Current Traits. 15](#_Toc194675064)

[3. Results After Excluding Participants Awaiting ASD Assessment in Study 1 16](#_Toc194675065)

[3.1 Developmental Trajectories of Autism Traits on Screening and Diagnostic Measures 16](#_Toc194675066)

[3.2 Emergence and Presentation of Autism Traits on Diagnostic Measures 19](#_Toc194675067)

[3.2.1 Early and Lifetime Traits. 19](#_Toc194675068)

[3.2.2 Current Traits. 20](#_Toc194675069)

[4. Results After Matching Groups for Assigned Sex at Birth and Age in Study 2 21](#_Toc194675070)

[4.1 Matching Procedure and Statistics 21](#_Toc194675071)

[4.1.1 Autism Diagnosis in Caregivers. 21](#_Toc194675072)

[4.1.2 Autism Traits in Caregivers. 21](#_Toc194675073)

[5. Results After Excluding Caregivers With ASD or Awaiting ASD Assessment in Study 2 23](#_Toc194675074)

[6. Deviations for Preregistration Study 1 25](#_Toc194675075)

[7. Deviations for Preregistration Study 2 26](#_Toc194675076)

# Results After Matching Groups for Age and FSIQ in Study 1

## 1.1 Developmental Trajectories of Autism Traits

### 1.1.1 Matching Procedure and Results on SCQ and AQ.

To ensure that the between-group differences reported in the manuscript resulted from differences in diagnostic status (autistic/nonautistic), rather than differences in age and FSIQ, we matched groups on these variables using an R script (version 4.3.3; R Core Team, 2024). Groups were considered matched if *p*-values were greater than .05 and effect sizes were small. To achieve this, we followed a series of steps.

Step 1: The youngest participants from the autistic cisgender, nonautistic cisgender, and nonautistic gender clinic-referred groups were gradually removed until sex ratio and group sizes (*n*s) were balanced with the autistic gender clinic-referred group. When participants within the same group had the same age, ties were resolved by excluding the participant with the lowest FSIQ from the autistic cisgender and nonautistic gender clinic-referred groups, and the participant with the highest FSIQ from the nonautistic cisgender group. After this step, analyses showed that the groups remained unmatched for age and FSIQ, so we proceeded to Step 2.

Step 2: The oldest assigned female at birth was excluded from the autistic gender clinic-referred group and the youngest female assigned at birth from each of the other groups. If multiple participants within the same group had the same age, ties were resolved as in Step 1. After this step, analyses showed that groups were matched for age but not for FSIQ, so we proceeded to Step 3.

Step 3: The participant with the highest FSIQ from the nonautistic cisgender group and those with the lowest FSIQ from the other groups, regardless of age or assigned sex at birth, were excluded. After this step, analyses showed that groups were matched for FSIQ, age and assigned sex at birth. The final matched sample characteristics and matching statistics are reported in Table S1.

| **Table S1** | | | | | | | | | | | |
| --- | --- | --- | --- | --- | --- | --- | --- | --- | --- | --- | --- |
| *Sample Characteristics and Matching Statistics on Age and FSIQ for SCQ and AQ Analysis in Study 1* | | | | | | | | | | | |
| Variable | Autistic | | Nonautistic | | | ANOVA | | | | | Direction of Effects |
|  | GC-referred | Cisgender | | GC-referred | Cisgender |  |  |  |  |  |  |
|  | *n* = 47  (47% AFAB)^a^ | *n* = 47  (49% AFAB) | | *n* = 47  (49% AFAB) | *n* = 47  (49 % AFAB) |  |  |  |  |  |  |
|  | *M* (*SD*) | *M* (*SD*) | | *M* (*SD*) | *M* (*SD*) | Effect | *F* | *p* | 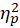 | BF_10_ |  |
| Age | 13.02 (2.28) | 12.00 (1.89) | | 12.40 (1.75) | 12.40 (1.65) | D | 0.15 | .703 | .00 | 0.17 | Autistic = Nonautistic |
|  |  |  | |  |  | G | 3.37 | .068 | .02 | 0.75 | GC-referred = Cisgender |
|  |  |  | |  |  | D × G | 3.37 | .068 | .02 | 0.99 |  |
|  |  |  | |  |  |  |  |  |  |  |  |
| FSIQ | 105.28 (13.34) | 103.40 (14.88) | | 102.98 (10.97) | 107.98 (11.65) | D | 0.37 | .543 | .00 | 0.19 | Autistic = Nonautistic |
|  |  |  | |  |  | G | 0.70 | .403 | .00 | 0.22 | GC-referred = Cisgender |
|  |  |  | |  |  | D × G | 3.39 | .067 | .02 | 1.25 |  |
|  |  |  | |  |  |  |  |  |  |  |  |
| Voc (*t* score) | 53.66 (8.60) | 51.21 (9.38) | | 53.47 (7.45) | 55.70 (7.34) | D | 3.20 | .075 | .02 | 0.69 | Autistic = Nonautistic |
|  |  |  | |  |  | G | 0.01 | .930 | .00 | 0.16 | GC-referred = Cisgender |
|  |  |  | |  |  | D × G | 3.80 | .053 | .02 | 1.21 |  |
|  |  |  | |  |  |  |  |  |  |  |  |
| MR (*t* score) | 52.51 (8.62) | 52.77 (10.41) | | 50.15 (8.85) | 53.57 (8.37) | D | 0.34 | .559 | .00 | 0.19 | Autistic = Nonautistic |
|  |  |  | |  |  | G | 1.92 | .167 | .01 | 0.39 | GC-referred = Cisgender |
|  |  |  | |  |  | D × G | 1.43 | .234 | .01 | 0.54 |  |
| *Note.* *N* = 188. AFAB = assigned female at birth; ANOVA = analysis of variance; FSIQ = full scale IQ-2; Voc = vocabulary subtest; MR = matrix reasoning subtest; GC-referred = gender clinic-referred; D = diagnostic status; G = gender identity status; SCQ = Social Communication Questionnaire – Lifetime; AQ = Autism-spectrum Quotient-Child/Adolescent.  ^a^ all *p*s related to assigned sex at birth ≥ .884 (BF_10_ = 0.09). | | | | | | | | | | | |

Table S2 shows the mean scores of autism traits *in each group* over time, measured by questionnaires, and the results of a three-way ANOVA. As in the original unmatched sample, a significant main effect of diagnostic status was found, reflecting significantly higher autism traits among autistic youth. Neither the main effect of gender identity status, nor the main effect of developmental timepoint were significant. All interactions were also nonsignificant.

| **Table S2** | | | | | | | | | | |
| --- | --- | --- | --- | --- | --- | --- | --- | --- | --- | --- |
| *Means, Standard Deviations, and Three-Way ANOVA Statistics for Caregiver-Reported Autism Traits (Z Scores) in Study 1* | | | | | | | | | | |
| Variable | Autistic | | Nonautistic | | ANOVA | | | | | Direction of Effects |
|  | GC-referred | Cisgender | GC-referred | Cisgender |  |  |  |  |  |  |
|  | *M (SD)* | *M (SD)* | *M (SD)* | *M (SD)* | Effect | *F* | *p* | 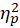 | BF_10_ |  |
| Autism traits |  |  |  |  |  |  |  |  |  |  |
| Early (SCQ) | 0.75 (0.95) | 0.76 (0.77) | -0.61 (0.58) | -0.68 (0.47) | D | 276.44 | <.001 | .60 | >100 | Autistic > Nonautistic |
| Current (AQ) | 0.86 (0.50) | 0.77 (0.60) | -0.58 (0.86) | -0.67 (0.63) | G | 0.54 | .462 | .00 | 0.29 | GC-referred = Cisgender |
|  |  |  |  |  | T | 0.59 | .443 | .00 | 0.15 | Early = Current |
|  |  |  |  |  | D × G | 0.08 | .782 | .00 | 0.25 |  |
|  |  |  |  |  | T × G | 0.31 | .581 | .00 | 0.18 |  |
|  |  |  |  |  | T × D | 0.13 | .714 | .00 | 0.18 |  |
|  |  |  |  |  | T × D × G | 0.16 | .689 | .00 | 0.24 |  |
| *Note.* *N* = 188. ANOVA = analysis of variance; D = diagnostic status; G = gender identity status; T = timepoint; GC-referred = gender clinic-referred; SCQ = Social Communication Questionnaire-Lifetime; AQ = Autism-Spectrum Quotient-Child/Adolescent. | | | | | | | | | | |

### 1.1.2 Matching Procedure and Results on ADI-R and BOSA.

To ensure that the nonsignificant between-group differences reported in the manuscript did not result from differences in age and FSIQ, we matched groups on these variables. To achieve this, the youngest participants from the autistic cisgender group were gradually removed until sex ratio and group size (*n*) were balanced with the autistic gender clinic-referred group. When participants within the same group had the same age, ties were resolved by excluding the participant with the lowest FSIQ from the autistic cisgender group. The final matched sample characteristics and matching statistics are reported in Table S3.

| **Table S3** | | | | | | | |
| --- | --- | --- | --- | --- | --- | --- | --- |
| *Sample Characteristics and Matching Statistics on Age and FSIQ for ADI-R and BOSA Analysis in Study 1* | | | | | | | |
| Variable | Autistic GC-referred | Autistic cisgender | *t*-test | | | | Direction of Effects |
|  | *n* = 41 (49% AFAB) | *n* = 41 (49% AFAB) |  |  |  |  |  |
|  | *M* (*SD*) | *M* (*SD*) | *t* | *p* | *d* | BF_10_ |  |
| Age | 12.95 (2.46) | 12.27 (1.78) | 1.44 | .153 | 0.31 | 0.57 | Autistic = Nonautistic |
|  |  |  |  |  |  |  |  |
| FSIQ | 105.15 (13.49) | 103.20 (15.31) | 0.61 | .542 | 0.14 | 0.27 | Autistic = Nonautistic |
|  |  |  |  |  |  |  |  |
| Voc (*t* score) | 53.51 (8.43) | 51.37 (9.80) | 1.06 | .291 | 0.24 | 0.38 | Autistic = Nonautistic |
|  |  |  |  |  |  |  |  |
| MR (*t* score) | 52.49 (8.77) | 52.37 (10.48) | 0.06 | .955 | 0.01 | 0.23 | Autistic = Nonautistic |
| Note. *N* = 82. AFAB = assigned female at birth; FSIQ = full scale IQ-2; Voc = vocabulary subtest; MR = matrix reasoning subtest; GC-referred = gender clinic-referred. | | | | | | | |

Table S4 shows the mean scores of social-communication abilities *in autistic participants only* over time and the results of a 2 × 2 ANOVA. As in the original unmatched sample, neither the main effect of gender identity status, nor the main effect of developmental timepoint were significant. The 2 × 2 interaction was also nonsignificant.

| **Table S4** | | | | | | | | |
| --- | --- | --- | --- | --- | --- | --- | --- | --- |
| *Means, Standard Deviations, and Two-Way ANOVA Statistics for Autism Traits (Z Scores) Assessed with Diagnostic Tools After Matching Groups for Age and FSIQ in Study 1* | | | | | | | | |
| Variable | Autistic GC-referred | Autistic cisgender | ANOVA | | | | | Direction of Effects |
|  | *M* (*SD*) | *M* (*SD*) | Effect | *F* | *p* | 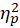 | BF_10_ |  |
| Autism traits |  |  |  |  |  |  |  |  |
| Early (ADI-R) | -0.09 (1.14) | 0.00 (0.93) | G | 0.00 | .976 | .00 | 0.20 | GC-referred = Cisgender |
| Current (BOSA) | 0.01 (0.97) | 0.10 (1.00) | T | 0.00 | .984 | .00 | 0.17 | Early = Current |
|  |  |  | T × G | 0.42 | .520 | .01 | 0.27 |  |
| *Note.* *N* = 82 (*n* = 41 for each group). ANOVA = analysis of variance; G = gender identity status; T = timepoint; ADI-R = Autism Diagnostic Interview- Revised (domains A+B composite score); BOSA = Brief Observation of Autism (domain A scores); GC-referred = gender clinic-referred. | | | | | | | | |

## 1.2 Emergence and Presentation of Autism Traits on Diagnostic Measures

### 1.2.1 Matching Procedure and Results on ADI-R.

To ensure that the nonsignificant between-group differences reported in the manuscript did not result from differences in age and FSIQ, we matched groups on these variables. The matching procedure followed the one described in 1.1.2. The final matched sample characteristics and matching statistics are reported in Table S5.

| **Table S5** | | | | | | | |
| --- | --- | --- | --- | --- | --- | --- | --- |
| *Sample Characteristics and Matching Statistics for Age and FIQ for ADI-R Analyses in Study 1* | | | | | | | |
| Variable | Autistic GC-referred | Autistic cisgender | *t*-test | | | | Direction of Effects |
|  | *n* = 45  (49% AFAB) | *n* = 45  (49% AFAB) |  |  |  |  |  |
|  | *M* (*SD*) | *M* (*SD*) | *t* | *p* | *d* | BF_10_ |  |
| Age | 12.89 (2.36) | 12.16 (1.78) | 1.66 | .100 | 0.35 | 0.74 | Autistic = Nonautistic |
|  |  |  |  |  |  |  |  |
| FSIQ-2 | 104.04 (13.51) | 102.24 (15.29) | 0.59 | .556 | 0.13 | 0.26 | Autistic = Nonautistic |
|  |  |  |  |  |  |  |  |
| Voc (*t* score) | 53.04 (8.25) | 50.87 (9.62) | 1.15 | .252 | 0.24 | 0.40 | Autistic = Nonautistic |
|  |  |  |  |  |  |  |  |
| MR (*t* score) | 51.69 (9.00) | 51.78 (10.65) | -0.04 | .966 | 0.01 | 0.22 | Autistic = Nonautistic |
| Note. *N* = 90. AFAB = assigned female at birth; FSIQ-2 = full scale IQ-2; Voc = vocabulary subtest; MR = matrix reasoning subtest; ADI-R = Autism Diagnostic Interview-Revised; GC-referred = gender clinic-referred. | | | | | | | |

In the autistic gender clinic-referred group, 34/45 participants (75.56%) scored above the cut-off on ADI-R Domain D, compared to 37/45 participants (82.22%) in the nonautistic group. As in original unmatched sample, a chi-square test revealed a nonsignificant between-group difference in the proportion of participants who manifested developmental atypicalities or at before the age of 3 years, χ²(1, *N* = 90) = 0.60, *p* = .438, *φ* = -.08, BF_10_ = 0.28.

Figure S1 shows mean scores in each autism group on domains A, B, and C of the ADI-R. As in the original unmatched sample, a MANOVA indicted no overall group differences across ADI-R domains, Pillai’s Trace = 0.01, *F*(3, 86) = 0.34, *p* = .796, BF_10_ = 0.01.

**Figure S1**

*ADI-R Scores for Autistic Gender Clinic-Referred and Autistic Cisgender Participants Across Domains After Matching Groups for FSIQ and Age*

*Note. N* = 90 (*n* = 45 for each group). Domain A **=** Reciprocal Social Interactions; Doman B = Communication; Domain C = Restricted, Repetitive, and Stereotyped Patterns of Behaviour; GC-referred = gender-clinic referred. Error bars show standard errors.

### 1.2.2 Matching Procedure and Results on BOSA.

Matching procedure and participant characteristic are identical to the ones described in 1.2. As in the original unmatched sample, the difference in the BOSA total score between autistic gender clinic-referred (*n* = 41, *M* = 8.49, *SD* = 2.79) and autistic cisgender participants (*n* = 41, *M* = 8.82, *SD* = 2.82) was nonsignificant, *t*(80) = -0.55 *p* = .583, *d* = 0.12, BF_10_ = 0.26. Furthermore, consistent with the results from the unmatched sample, autistic gender clinic-referred participants showed fewer current restricted interests and behaviours on domain B (*n* = 41, *M* = 1.71, *SD* = 1.21) than autistic cisgender participants (*n* = 41, *M* = 2.29, *SD* = 1.01), *t*(80) = -2.38, *p* = .020, *d* = 0.52, BF_10_ = 2.61.

# 2. Results After Excluding Participants Who Identified With Their Assigned Sex at Birth From the Gender Clinic-Referred Group in Study 1

## 2.1 Developmental Trajectories of Autism Traits on Screening and Diagnostic Measures

Table S6 shows the mean scores of autism traits *in each group* over time, measured by questionnaires (i.e., SCQ and AQ), and the results of a three-way ANOVA after excluding gender clinic-referred participants whose expressed gender aligned with their assigned sex at birth (*n* = 5) from the original unmatched sample. As in the original sample, a significant main effect of diagnostic status was found, reflecting significantly higher autism traits among autistic youth. Neither the main effect of gender identity status, nor the main effect of developmental timepoint were significant. All interactions were also nonsignificant.

Table S7 shows the mean scores of social-communication abilities, measured by diagnostic tools (i.e., ADI-R and BOSA) *in autistic participants only* over time and the results of a 2 × 2 ANOVA after excluding gender clinic-referred participants whose expressed gender aligned with their assigned sex at birth (*n* = 3) from the original unmatched sample. As in the original sample, neither the main effect of gender identity status, nor the main effect of developmental timepoint were significant. The interaction was also nonsignificant.

| **Table S6** | | | | | | | | | | |
| --- | --- | --- | --- | --- | --- | --- | --- | --- | --- | --- |
| *Means, Standard Deviations, and Three-Way ANOVA Statistics for Caregiver-Reported Autism Traits (Z Scores) in Study 1* | | | | | | | | | | |
| Variable | Autistic | | Nonautistic | | ANOVA | | | |  | Direction of Effects |
|  | GC-referred | Cisgender | GC-referred | Cisgender |  |  |  |  |  |  |
|  | *M (SD)* | *M (SD)* | *M (SD)* | *M (SD)* | Effect | *F* | *p* | 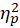 | BF_10_ |  |
| Autism traits |  |  |  |  |  |  |  |  |  |  |
| Early (SCQ) | 0.69 (0.96) | 0.81 (0.78) | -0.64 (0.54) | -0.70 (0.46) | D | 362.79 | <.001 | .62 | >100 | Autistic > Nonautistic |
| Current (AQ) | 0.85 (0.50) | 0.77 (0.58) | -0.67 (0.83) | -0.74 (0.64) | G | 0.10 | .757 | .00 | 0.25 | GC-referred = Cisgender |
|  |  |  |  |  | T | 0.10 | .759 | .00 | 0.11 | Early = Current |
|  |  |  |  |  | D × G | 0.26 | .612 | .00 | 0.24 |  |
|  |  |  |  |  | T × G | 1.27 | .262 | .01 | 0.26 |  |
|  |  |  |  |  | T × D | 1.09 | .298 | .01 | 0.24 |  |
|  |  |  |  |  | T × D × G | 0.89 | .345 | .00 | 0.27 |  |
| *Note.* *N* = 224 (*n* = 46 for autistic gender clinic-referred group; *n* = 57 for autistic cisgender group; *n* = 54 for nonautistic gender clinic-referred group; *n* = 67 for nonautistic cisgender group). ANOVA = analysis of variance; D = diagnostic status; G = gender identity status; T = timepoint; SCQ = Social Communication Questionnaire-Lifetime; AQ = Autism-spectrum Quotient-Child/Adolescent; GC-referred = gender clinic-referred. | | | | | | | | | | |

| **Table S7** | | | | | | | | |
| --- | --- | --- | --- | --- | --- | --- | --- | --- |
| *Means, Standard Deviations, and Two-Way ANOVA Statistics for Autism Traits (Z Scores) Assessed with Diagnostic Tools in Study 1* | | | | | | | | |
| Variable | Autistic | | ANOVA | | | |  | Direction of Effects |
|  | GC-referred | Cisgender |  |  |  |  |  |  |
|  | *M* (*SD*) | *M* (*SD*) | Effect | *F* | *p* | 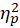 | BF_10_ |  |
| Autism Traits |  |  |  |  |  |  |  |  |
| Early (ADI-R) | -0.16 (1.16) | 0.07 (0.88) | G | 0.54 | .465 | .01 | 0.23 | GC-referred = Cisgender |
| Current (BOSA) | 0.00 (1.00) | -0.01 (1.03) | T | 0.07 | .792 | .00 | 0.16 | Early = Current |
|  |  |  | T × G | 0.64 | .426 | .01 | 0.31 |  |
| *Note.* *N* = 91 (*n* = 38 for autistic gender clinic-referred group; *n* = 53 for autistic cisgender group). ANOVA = analysis of variance; G = gender identity status; GC-referred = gender clinic-referred; T = timepoint; ADI-R = Autism Diagnostic Interview- Revised (domains A+B composite score); BOSA = Brief Observation of Autism (domain A scores). | | | | | | | | |

## 2.2 Emergence and Presentation of Autism Traits on Diagnostic Measures

### 2.2.1 Early and Lifetime Traits.

In the autistic gender clinic-referred group, 31/42 participants (73.81%) scored above the cut-off on ADI-R Domain D, compared to 49 out of 57 (85.96%) in the autistic cisgender group. As in the original sample, a chi-square test revealed a small and nonsignificant between-group difference in the proportion of participants who manifested developmental atypicalities at or before the age of 3 years, χ²(1, *N* = 99) = 2.30, *p* = .129, *φ* = -.15, BF_10_ = 0.94.

Figure S2 shows mean scores in each autism group on domains A, B, and C of the ADI-R. As in the original sample, a MANOVA indicted no overall group differences across ADI-R domains, Pillai’s Trace = 0.05, *F*(3, 95) = 1.55, *p* = .207, BF_10_ = 0.15.

**Figure S2**

*ADI-R Scores for Autistic Gender Clinic-referred and Autistic Cisgender Participants Across Domains*

*Note. N* = 99 (*n* = 42 for autistic gender clinic-referred group; *n* = 57 for autistic cisgender group). Domain A **=** Reciprocal Social Interactions; Doman B = Communication; Domain C = Restricted, Repetitive, and Stereotyped Patterns of Behaviour; GC-referred = gender clinic referred. Error bars show standard errors.

### 2.2.2 Current Traits.

As in the original sample, there difference in the BOSA total score between autistic gender clinic-referred (*n* = 38, *M* = 8.47, *SD* = 2.85) and autistic cisgender (*n* = 53, *M* = 8.98, *SD* = 2.76) participants was nonsignificant, *t*(89) = 0.85, *p* = .396, *d* = 0.18, BF_10_ = 0.31**.** Furthermore, consistent with the results from the original sample, autistic gender clinic-referred participants showed fewer current restricted interests and behaviours on domain B (*M* = 1.71, *SD* = 1.23) than autistic cisgender participants (*M* = 2.25, *SD* = 1.13), *t*(89) = 2.15, *p* = .034, *d* = 0.46, BF_10_ = 1.66.

# 3. Results After Excluding Participants Awaiting ASD Assessment in Study 1

## 3.1 Developmental Trajectories of Autism Traits on Screening and Diagnostic Measures

Table S8 shows the mean scores of autism traits *in each group* over time, measured by questionnaires (i.e., SCQ and AQ), and the results of a three-way ANOVA after excluding participants who were awaiting an ASD assessment (*n* = 8) from the original sample. As in the original sample, a significant main effect of diagnostic status was found, reflecting significantly higher autism traits among autistic than nonautistic youth. Neither the main effect of gender identity status, nor the main effect of developmental timepoint were significant. All interactions were also nonsignificant.

Table S9 shows the mean scores of social-communication abilities *in autistic participants only* over time, measured with diagnostic tools (i.e., ADI-R and BOSA), and the results of a 2 × 2 ANOVA after excluding participants who were awaiting an ASD assessment (*n* = 6) from the original sample. As in the original sample, neither the main effect of gender identity status, nor the main effect of developmental timepoint were significant. The interaction was also nonsignificant.

| **Table S8** | | | | | | | | | | | |
| --- | --- | --- | --- | --- | --- | --- | --- | --- | --- | --- | --- |
| *Means, Standard Deviations, and Three-Way ANOVA Statistics for Caregiver-Reported Autism Traits (Z Scores) in Study 1* | | | | | | | | | | | |
| Variable | Autistic | | Nonautistic | | ANOVA | | | |  | Direction of Effects | |
|  | GC-referred | Cisgender | Gender-referred | Cisgender |  |  |  |  |  |  |  |
|  | *M (SD)* | *M (SD)* | *M (SD)* | *M (SD)* | Effect | *F* | *p* | 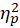 | BF_10_ |  |  |
| Autism traits |  |  |  |  |  |  |  |  |  |  |  |
| Early (SCQ) | 0.96 (0.87) | 0.81 (0.78) | -0.64 (0.54) | -0.70 (0.46) | D | 427.71 | <.001 | .66 | >100 | Autistic > Nonautistic |  |
| Current (AQ) | 0.95 (0.40) | 0.77 (0.58) | -0.66 (0.83) | -0.74 (0.64) | G | 2.45 | .119 | .01 | 0.28 | Gender clinic-referred = Cisgender |  |
|  |  |  |  |  | T | 0.29 | .588 | .00 | 0.12 | Early = Current |  |
|  |  |  |  |  | D × G | 0.43 | .515 | .00 | 0.27 |  |  |
|  |  |  |  |  | T × G | 0.08 | .785 | .00 | 0.14 |  |  |
|  |  |  |  |  | T × D | 0.02 | .889 | .00 | 0.16 |  |  |
|  |  |  |  |  | T × D × G | 0.00 | .966 | .00 | 0.19 |  |  |
| *Note.* *N* = 221. ANOVA = analysis of variance; D = diagnostic status; G = gender identity status; T = timepoint; SCQ = Social Communication Questionnaire – Lifetime; AQ = Autism-spectrum Quotient-Child/Adolescent. | | | | | | | | | | | |

| **Table S9** | | | | | | | | |
| --- | --- | --- | --- | --- | --- | --- | --- | --- |
| *Means, Standard Deviations, and Two-Way ANOVA Statistics for Autism Traits (Z Scores) Assessed with Diagnostic Tools in Study 1* | | | | | | | | |
| Variable | Autistic | | ANOVA | | | |  | Direction of Effects |
|  | GC-referred | Cisgender |  |  |  |  |  |  |
|  | *M* (*SD*) | *M* (*SD*) | Effect | *F* | *p* | 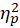 | BF_10_ |  |
| Autism Traits |  |  |  |  |  |  |  |  |
| Early (ADI-R) | 0.10 (1.02) | 0.07 (0.88) | G | 0.00 | .950 | .00 | 0.19 | GC-referred = Cisgender |
| Current (BOSA) | -0.06 (1.03) | -0.01 (1.03) | T | 0.62 | .433 | .01 | 0.22 | Early = Current |
|  |  |  | T × G | 0.06 | .804 | .00 | 0.25 |  |
| *Note.* *N* = 88 (*n* = 35 for autistic gender clinic-referred group; *n* = 53 for autistic cisgender group). ANOVA = analysis of variance; G = gender identity status; T = timepoint; GC-referred = gender clinic-referred; ADI-R = Autism Diagnostic Interview-Revised (domains A+B composite score); BOSA = Brief Observation of Autism (domain A scores). | | | | | | | | |

## 3.2 Emergence and Presentation of Autism Traits on Diagnostic Measures

### 3.2.1 Early and Lifetime Traits.

In the autistic gender clinic-referred group, 29/38 participants (76.32%) scored above the cut-off on ADI-R Domain D, compared to 49 out of 57 (85.96%) in the autistic cisgender group. As in the original sample, a chi-square test revealed a small and nonsignificant between-group difference in the proportion of participants who manifested developmental atypicalities at or before the age of 3 years, χ²(1, *N* = 95) = 1.45, *p* = .229, *φ* = -.12, BF_10_ = 0.64.

Figure S3 shows mean scores in each autism group on domains A, B, and C of the ADI-R. As in the original sample, a MANOVA indicted no overall group differences across ADI-R domains, Pillai’s Trace = 0.01, *F*(3, 91) = 0.24, *p* = .871, BF_10_ = 0.01.

*Note. N* = 95 (*n* = 38 for autistic gender clinic-referred group; *n* = 57 for autistic cisgender group). Domain A **=** Reciprocal Social Interactions; Doman B = Communication; Domain C = Restricted, Repetitive, and Stereotyped Patterns of Behaviour; GC-referred = gender clinic-referred. Error bars show standard errors.

**Figure S3**

*ADI-R Scores for Autistic Gender Clinic-Referred and Autistic Cisgender Participants Across Domains*

### 3.2.2 Current Traits.

As in the original sample, there was a nonsignificant difference between autistic gender clinic-referred (*n* = 35, *M* = 8.20, *SD* = 2.84) and autistic cisgender (*n* = 53, *M* = 8.98, *SD* = 2.76) participants in the BOSA total score, *t*(86) = 1.28, *p* = .202, *d* = 0.28, BF_10_ = 0.47. Furthermore, consistent with the results from the original sample, autistic gender clinic-referred participants showed fewer current restricted interests and behaviours on domain B (*M* = 1.60, *SD* = 1.19) than autistic cisgender participants (*M* = 2.25, *SD* = 1.13), *t*(86) = 2.57, *p* = .012, *d* = 0.56, BF_10_ = 3.85.

# 4. Results After Matching Groups for Assigned Sex at Birth and Age in Study 2

## 4.1 Matching Procedure and Statistics

To achieve this matching, the youngest caregivers from each group were gradually removed, separately for assigned males at birth and assigned females at birth, until groups were balanced in *n* with the group of caregivers of autistic gender clinic-referred youth. Next, the youngest participant from each of the cisgender groups and the oldest participants from each of the gender clinic-referred groups were excluded. After this procedure, groups were matched for both age and assigned sex at birth (see Table S10).

### 4.1.1 Autism Diagnosis in Caregivers.

A series of one-proportion *z*-tests compared the proportion of ASD diagnoses in each group of caregivers to the general population prevalence (1%). As in the original unmatched sample, the results showed that the proportion of ASD diagnoses was significantly higher in caregivers of autistic gender clinic-referred (7.84%) and autistic cisgender (7.84%) youth than in the general population, *z* = 4.90, *p* < .001, BF_10_ = 18.90 and *z* = 4.90, *p* < .001, BF_10_ = 18.90, respectively. None of the caregivers in the nonautistic groups, both gender clinic-referred and cisgender youth, reported a diagnosis of ASD, and this did not differ significantly from the population estimate, *z* = -0.71, *p* = .478, BF_10_ = 0.89 and *z* = -0.72, *p* = .472, BF_10_ = 0.89, respectively.

### 4.1.2 Autism Traits in Caregivers.

As in the original sample, a 2 × 2 ANOVA on caregiver AQ-50 score revealed a significant main effect of child diagnostic status, indicating that caregivers of autistic participants reported significantly more autism traits than caregivers of nonautistic children, regardless of their child’s gender identity status (see Table S10). Neither the main effect of child gender identity status nor the interaction was significant.

| **Table S10** | | | | | | | | | | |
| --- | --- | --- | --- | --- | --- | --- | --- | --- | --- | --- |
| *Sample Characteristics and Two-Way ANOVA Statistics for Age and Autism Traits in Study 2* | | | | | | | | | | |
| Variable | Autistic | | Nonautistic | | ANOVA | | | |  | Direction of Effects |
|  | GC-referred | Cisgender | GC-referred | Cisgender |  |  |  |  |  |  |
|  | *n* = 51  (98% AFAB) | *n* = 51  (98% AFAB) | *n* = 50  (98% AFAB) | *n* = 51  (98% AFAB) |  |  |  |  |  |  |
|  | *M* (*SD*) | *M* (*SD*) | *M* (*SD*) | *M* (*SD*) | Effect | *F* | *p* | 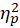 | BF_10_ |  |
| Age | 44.96 (4.64) | 43.18 (6.34) | 43.92 (6.27) | 43.39 (4.48) | D | 0.29 | .594 | .00 | 0.18 | Autistic = Nonautistic |
|  |  |  |  |  | G | 2.24 | .136 | .01 | 0.44 | GC-referred = Cisgender |
|  |  |  |  |  | D × G | 0.66 | .417 | .00 | 0.30 |  |
|  |  |  |  |  |  |  |  |  |  |  |
| Autism traits (AQ) | 20.47 (12.01) | 21.76 (11.67) | 14.50 (8.71) | 15.43 (7.15) | D | 18.83 | <.001 | .09 | >100 | Autistic > Nonautistic |
|  |  |  |  |  | G | 0.62 | .433 | .00 | 0.20 | GC-referred = Cisgender |
|  |  |  |  |  | D × G | 0.02 | .898 | .00 | 0.21 |  |
| *Note.* *N* = 226. AFAB = assigned female at birth; ANOVA = analysis of variance; D = child diagnostic status; G = child gender identity status; AQ = Autism-spectrum Quotient; GC-referred = gender clinic-referred. | | | | | | | | | | |

# 5. Results After Excluding Caregivers With ASD or Awaiting ASD Assessment in Study 2

A 2 (child diagnostic status: autistic/nonautistic) 2 (child gender identity status: gender-referred/cisgender) ANOVA was conducted on caregiver AQ-50 score after excluding caregivers with, or awaiting assessment for, a diagnosis of ASD. As in the original sample, a 2 × 2 ANOVA revealed a significant main effect of child diagnostic status, indicating that *nonautistic* caregivers of autistic participants reported significantly more autism traits than caregivers of nonautistic children, regardless of their child’s gender identity status (see Table S11). Neither the main effect of child gender identity status nor the Child Gender Identity Status × Child Diagnostic Status interaction was significant.

| **Table S11** | | | | | | | | | | | |
| --- | --- | --- | --- | --- | --- | --- | --- | --- | --- | --- | --- |
| *Sample Characteristics and Two-Way ANOVA Statistics for Age and Autism Traits in Study 2* | | | | | | | | | | | |
| Variable | Autistic | | Nonautistic | | ANOVA | | | |  | Direction of Effects |  |
|  | GC-referred | Cisgender | GC-referred | Cisgender |  |  |  |  |  |  |  |
|  | *n* = 44  (98% AFAB) | *n* = 48  (92% AFAB) | *n* = 54  (91% AFAB) | *n* = 63  (81% AFAB) |  |  |  |  |  |  |  |
|  | *M* (*SD*) | *M* (*SD*) | *M* (*SD*) | *M* (*SD*) | Effect | *F* | *p* | 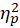 | BF_10_ |  |  |
| Autism traits (AQ) | 17.98 (11.14) | 18.77 (9.30) | 14.52 (8.79) | 15.59 (6.59) | D | 7.17 | .008 | .03 | 4.16 | Autistic > Nonautistic |  |
|  |  |  |  |  | G | 0.56 | .454 | .00 | 0.19 | GC-referred = Cisgender |  |
|  |  |  |  |  | D × G | 0.01 | .912 | .00 | 0.21 |  |  |
| *Note.* *N* = 209. AFAB = assigned female at birth; ANOVA = analysis of variance; D = child diagnostic status; G = child gender identity status; AQ = Autism-spectrum Quotient; GC-referred = gender clinic-referred. | | | | | | | | | | | |

# 6. Deviations for Preregistration Study 1

| **Where?** | **What?** | **Why?** |
| --- | --- | --- |
| Sample size | The final sample consisted of 49 autistic gender-referred, 57 autistic cisgender, 56 nonautistic gender-referred, and 67 nonautistic cisgender participants rather than 50 participants in each group. | - The insufficient number of autistic participants in the ongoing longitudinal study, which was the main recruitment source for gender clinic-referred participants, along with the closure of the only national gender identity service in the UK, and challenges reaching this population through other means, explain why the target sample size for the autistic gender clinic-referred group was not achieved. - To maximise statistical power, additional nonautistic gender-referred, nonautistic cisgender, and autistic cisgender groups participants recruited for matching purposes were included in the analysis. Results for the matched groups are reported in the Supplementary Information. |
| Data collection procedures | - Age range 7–16 years instead of 7–14 years - Use of social media to recruit both cisgender and gender clinic-referred participants | To maximise the chances of achieving our target sample size, and consequently, ensure we have adequate statistical power to conduct our planned statistical analyses. |
| Data exclusion | - Gender clinic-referred children undergoing an ASD assessment were included in the autistic gender clinic-referred group (*n* = 8). - Gender clinic-referred children who reported an expressed gender that aligned with their assigned sex at birth were included in the gender clinic-referred group (*n* = 5). | Due to difficulties meeting our target sample size for the gender clinic-referred groups. When these participants were excluded from the sample, results did not change substantively. |

# 7. Deviations for Preregistration Study 2

| **Where?** | **What?** | **Why?** |
| --- | --- | --- |
| Sample size | The final sample consisted of caregivers of 52 autistic gender-referred, 56 autistic cisgender, 55 nonautistic gender referred, and 63 nonautistic cisgender youth, rather than 50 participants in each group. | To maximise statistical power, additional participants recruited for matching purposes were included in the analysis. Results for the matched groups are reported in the Supplementary Information. |
| Data analysis | Between-group differences in sex ratio were examined conducting a series of Fisher’s exact tests, rather than a 3-way loglinear analysis. | Due to the small number of assigned male at birth caregivers in the sample, there were expected cell counts below 5. |
